# Anger’s moderating influence on the relationship between victimization and perpetration of domestic violence and abuse in patients suffering from severe mental illness. Insights from a cross sectional study using moderated mediation analysis

**DOI:** 10.3389/fpsyt.2024.1509982

**Published:** 2024-12-24

**Authors:** Roos Eva Ruijne, Milan Zarchev, Jens Henrichs, Carlo Garofalo, Stefan Bogaerts, Cornelis Lambert Mulder, Astrid Kamperman

**Affiliations:** ^1^ Department of Psychiatry, Erasmus Medical Center, Rotterdam, Netherlands; ^2^ Community Mental Healthcare, Parnassia, The Hague, Netherlands; ^3^ Department of Midwifery Science, AVAG and the EMGO+ Institute for Health and Care Research, Vrije Universtiteit (VU) Amsterdam, Amsterdam, Netherlands; ^4^ Amsterdam Public Health Research Institute, VU Medical Center, Amsterdam, Netherlands; ^5^ Department of Philosophy, Social Sciences, Humanities and Education, University of Perugia, Perugia, Italy; ^6^ Department of Social Psychology, Tilburg School of Social and Behavioral Sciences, Tilburg University, Tilburg, Netherlands; ^7^ Epidemiological and Social Psychiatric Research Institute, Department of Psychiatry, Erasmus Medical Center, Rotterdam, Netherlands; ^8^ Antes, Department of the Parnassia Psychiatric Institute, Rotterdam, Netherlands

**Keywords:** domestic violence and abuse, victimization, perpetration, severe mental illness, interpersonal violence, cross-sectional study

## Abstract

**Introduction:**

Domestic violence and abuse (DVA) are prevalent among persons with severe mental illness (SMI), being involved as victim, perpetrator, or both.

**Aims:**

To assess rates of DVA victimization and perpetration in patients with SMI. We also aimed to assess whether DVA victimization was associated with DVA perpetration, and whether this was mediated by dispositional anger in patients with SMI. Lastly, we aimed to examine whether gender moderated the associations between DVA victimization and perpetration.

**Methods:**

We conducted a nation-wide survey on victimization in patients with SMI. In 942 patients DVA perpetration of physical assault and victimization of physical assault, sexual coercion or psychological aggression over the past year were assessed using the revised Conflict Tactics Scale. Anger was assessed using the dispositional anger reactions scale. Correlation and mediation analyses were conducted, followed by a moderated mediation to assess whether effects of anger differed between men and women.

**Results:**

The prevalence rate of perpetration of physical assault was 22%, for victimization 27% and 52% for both. We found a strong positive correlation between perpetrated physical assault and victimization of mild physical assault and between both the perpetration and victimization of severe physical assault. Anger mediated the link between being a victim of psychological aggression and being a perpetrator of DVA. Women were more likely to perpetrate violence if they were victims of mild physical assault compared to men. Other moderation effects by gender were not observed.

**Conclusion and implications:**

This study reveals persistent high DVA rates among patients with SMI. Overall, anger had no mediating effect on the association between victimization and perpetration of violence, except for psychological aggression and perpetration of DVA. This study emphasizes the importance of routine violence discussions in SMI care while taking context into account. However, further research on underlying mechanisms and interventions to improve discussions and care for victims and/or perpetrators of DVA is necessary.

## Introduction

The World Health Organization (WHO) defines domestic violence and abuse (DVA) as behaviors within an intimate relationship that causes physical, sexual, or psychological harm (1). These behaviors include physical aggression, sexual coercion, psychological abuse and controlling behaviors (2). An intimate relationship could have a romantic character, but also a close relationship with a roommate, relative, family member or close friend can be defined as an intimate relationship. Experiencing DVA, whether as a victim or perpetrator, is associated with serious consequences for the physical and mental health of both individuals and families (3–6). Studies have found that the risk of having chronic medical conditions such as hypertension or traumatic brain injury in victims of DVA is increased (7–10). Victims of DVA also have a higher risk of developing a psychiatric disorder, including depression, anxiety and post-traumatic stress disorder (PTSD), with prevalence rates of mental illnesses in victims of DVA ranging from 5% to 67.2%. Studies have shown that victims of DVA are twice as likely to develop depression or PTSD (depression OR: 2.04-3.14, PTSD OR: 2.15-2.66) compared to non-victims (8, 11–15). In addition, perpetrators of DVA are more likely to suffer from suicidal behavior, substance abuse, depression and anxiety. Specific prevalence rates among perpetrators include 15.2% for panic disorders, 27.6% for social phobia, and 39.1% for alcohol use disorder, significantly exceeding general population rates (16–19). Moreover, patients suffering from severe mental illness (SMI) have an increased risk of being a victim of DVA compared to the general population (20–24). A systematic review and meta-analysis by Trevillion et al. (25) showed that 16.7% to 43.8% of individuals experienced DVA victimization in the past year. Research also shows that patients with a mental health disorder are more often a perpetrator of DVA in comparison to the general population with odd ratio’s ranging from 1.23 to 6.81 (26–29) with the odds being raised but lower for alcohol abuse and obsessive compulsive disorder and the odds of perpetration of IPV being the highest with personality disorders and mood disorders, including bipolar disorder and depressive disorders.

Perpetration of DVA is primarily attributed to men but there is increasing evidence of more gender symmetry in both victimization and perpetration of DVA than previously assumed (7, 30–33). Kivisto et al. (34) also suggest that women recently discharged from mental health clinics are more likely to commit DVA than men recently discharged. Conversely, while women are often portrayed as victims of DVA, there is increasing evidence that male SMI patients are also more likely to be victims of DVA than previously assumed (24, 35).

Victimization of DVA is also a common phenomenon, prevalence rates vary globally from 3% to 26% (24, 36). People with a SMI are 4 to 6 time more at risk of DVA (21, 25, 37) and are also more likely to commit DVA (26).

There are several theories on why certain people are more at risk to be victimized compared to others (33). One of these theories is risk heterogeneity, the believe that certain victims possess characteristics that make them likely targets for victimization and recurrent victimization (38). However, it is still unclear which characteristics add to this risk heterogeneity, especially when it comes to specific types of violence such as DVA (39, 40). Several studies have shown that, in addition to personality disorder and other forms of psychopathology, the level of anger could play a role in displaying aggression and anger is mentioned as one of the risk factors for committing DVA (39, 41). In addition, DVA victimization and perpetration often co-occur (32, 42), one explanation for this could be that victims of violence at one point retaliate and answer violence with –physical- violence (43).

Anger in this study it is defined as a reaction to a person’s assessment of injustice done to them by another person and/or institute (44). Anger varies in intensity and expression; it can range from being irritated to feeling furious (45). Research has shown that people who experienced intense anger more often are associated with committing DVA for both men and women (16, 42, 46). Anger does not necessarily translate into aggressive behavior. However, people who experience anger as a trait, also called dispositional anger, are also more likely to experience anger as a state, which may predict aggressive behavior (47, 48). Aggressive behavior can take many forms, ranging from overt physical violence to more subtle forms of aggression that may not be immediately recognizable as such. Of these different types of aggressive behavior, physical violence is typically the most easily identified by its victims and perpetrators. This could mean that surveys of perpetration of physical violence could be more reliable. Patients with SMI more often experience a higher state and trait of anger and are therefore also more likely to commit violence compared to the general population (41, 49, 50). In addition, higher levels of anger are also associated with a higher prevalence of victimization (33, 51, 52). Patients with SMI also often have a history of trauma in their childhood (53, 54). Having a history of traumatic events, particularly childhood abuse, could lead to higher levels of anger and consequently increased victimization and perpetration of DVA (55, 56). Lastly, research has found that overall, men and women tend to express anger differently (57). Women tend to express anger in internal ways and men in external ways, which affects in how they handle conflict. However, much is still unknown and gaining more knowledge about possible mechanisms linking victimization, perpetration and anger could help to provide crucial information for the development of interventions that seek to reduce victimization and perpetration of DVA in SMI patients.

**Figure 1:**
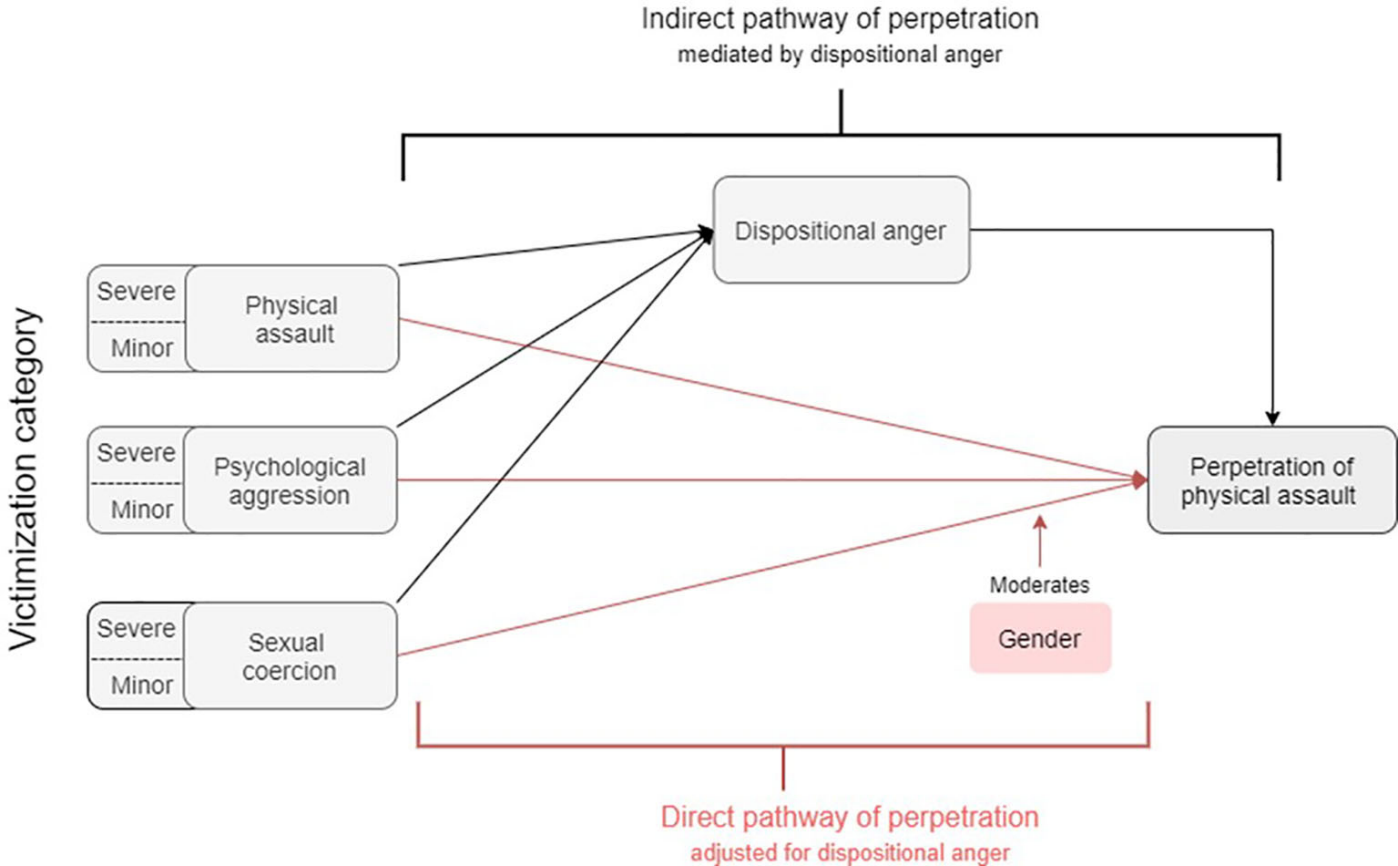
Moderated mediation model of the direct and indirect effects of victimization of physical assault, psychological aggression and sexual coercion on the perpetration of violence.

In this cross-sectional study, we first present rates of DVA from a victim and perpetration perspective in SMI patients. Second, we investigate pathways to perpetration of physical violence in patients with SMI. We hypothesize that victimization operates on perpetration through both a direct and an indirect pathway via anger. Furthermore, we want to examine whether (a) gender moderated the association between DVA victimization and perpetration and (b) whether it also moderated the mediation effects of anger on this link (see **Figure 1** for a conceptual model depicting our research aims).

## Materials and methods

### Participants

Adult patients (aged between 18 and 65 years) who met the criteria for SMI were eligible for participation. SMI is defined as schizophrenia, schizoaffective -, bipolar -, personality - or major depressive disorder according to the DSM IV- lasting at least two years and patients with SMI have significant limitations in social/societal functioning caused by having SMI (58). Recruitment took place through clinicians responsible for the treatment of the participants, providing outpatient care to patients with chronic (> 2 years duration) depressive, bipolar or psychotic disorders. Six MHC institutions participated and provided care to approximately 9250 patients. All patients who received care from these institutions were assessed for eligibility. Exclusion criteria were patients who were not proficient in the Dutch language and patients who were unable to answer questions or give consent for participation because of their psychiatric condition.

### Study design

This study is part of a larger cross-sectional study on victimization in psychiatric patients (37). This study was conducted between December 2011 and April 2012. The Medical Ethical Committee of the Erasmus Medical Centre approved this study (MEC-2010-232). All participants provided written informed consent prior to participation and could stop their participation at any time, without a given reason. The results were not traceable to the individual patient level.

### Procedure

First, a random sample of 3336 eligible patients was selected, for an in depth description of the sample selection, we refer to the study of Kamperman et al., 2014. Second, eligibility of each patient was cross validated by their primary mental healthcare clinician that resulted in a sample of 2,572 patients. These patients received a letter inviting them to participate in the study. After contact was made and informed consent obtained, the face-to-face interview was conducted by trained interviewers. A total of 1,046 patients were interviewed, of whom 942 completed all surveys for the current analysis. The interview contained global questions on personal information characteristics such as gender and age, and questions about topics such as victimization, discrimination, trauma and anger. Participants received a 20€ incentive for participation. Full details of the study design and interview instrument have been previously reported (37). **Supplementary Figure 1** provides a flowchart of the recruitment process.

### Measures

#### Domestic violence and abuse victimization and perpetration

The Revised Conflict Tactics Scale (CTS-2) (59) is a widely used questionnaire to detect victimization and perpetration of violence in the past year, in the context of romantic relationships. As mentioned in the introduction, we adapted the CTS-2, including other personal intimate relationships, such as family members, friends or roommates. Participants were instructed on this broader definition of DVA. The instrument consists of 39 items categorized by severity (minor or severe)and type (emotional or cognitive) across five subscales: negotiation (6 items: 3 emotional), psychological aggression (8 items: 4 minor, 4 severe), physical assault (12 items: 5 minor, 7 severe), sexual coercion (7 items: 3 minor, 4 severe), and injury (6 items: 2 minor and 4 severe). To assess victimization, we included the subscales psychological aggression, physical assault, and sexual coercion. To assess perpetration, we had data from the subscale physical assault. Mild psychological aggression comprises of insulting, swearing, yelling, shouting, or spiting; severe forms include threatening, accusing, or destroying an object belonging to the victim. Mild sexual coercion includes forcing someone to have sex without a condom or insisting on (certain forms of) sex without physical force, while severe forms are marked using threats or physical force. Shoving, pushing, grabbing or pulling are defined as minor forms of physical assault. Severe forms are choking, slamming, burning, kicking or using a knife or gun. Respondents were asked to rate the frequency of each incident of violence over the past 12 months using a 8-point Likert scale with the following categories: 0 ‘never’, 1 ‘one time’, 2 ‘two times’, 3 ‘three to five times’, 4 ‘six to ten times’, 5 ‘eleven to twenty times’, 6 ‘more than twenty times’, or 7 ‘this happened, but not in the past twelve months’. Since we were only interested in 12-month prevalence, we recoded the last category into 0. We calculated sum scores for the total subscale, and for minor and severe incidents separately. Subscale scores were dichotomized into presence or absence of violence with a cut-off score of 1 or higher. In line with the recommendations of the scale developers, we calculated the number of incidents per subscale by summing the midpoints of each response category. Twenty-five was used as the midpoint for the 20 times or higher-category (59). Psychometric qualities of the CTS-2 are considered to be well studied in a multitude of respondent samples. Internal consistency of the subscales was found to be good to very good ranging from 0.79 for psychological aggression to 0.95 for injury (59, 60).

#### Anger

Anger is assessed using the Dimensions of Anger (DAR) questionnaire (61). The DAR consists of 7 statements, representing anger frequency, intensity, duration, antagonistic expression, impairment of work performance due to anger, interpersonal relationships and anger, and personal health in relation to anger. Statements for example were ‘I often find myself getting angry at people or situations’ and ‘My anger prevents me from getting along with people as well as I’d like to’ (62). Respondents rate the extent to which these statements represent them on a 5-point Likert scale: from 0 ‘not at all’ to 4 ‘very much’. The total score can range from 0 to 28. A higher score corresponds with a higher tendency to respond with anger, or a higher dispositional anger. A mean item score of 2.4 or higher is considered to indicate pathological levels of dispositional anger (61). Previous literature showed that the DAR survey has good to excellent psychometric properties with Cronbach’s alphas ranging from 0.69-0.71 to 0.91 in earlier studies (61, 62). The scale showed a good internal consistency in this sample (Cronbach’s alpha: 0.79).

### Statistical analysis

Difference in demographic characteristics between men and women were tested using T-test or Mann-Whitney test for continuous variables (depending on the shape of their distribution), and with Chi2-test for categorical variables. Prevalence rates of victimization and perpetration and corresponding 95% confidence intervals are reported for the full sample, and for men and women separately. Differences between men and women in the dichotomized victimization and perpetration outcomes were tested using logistic regression analysis. We report the Odds Ratio (OR) and the 95% confidence interval (CI), where higher odds correspond to women the higher prevalence of the women in the sample. Since incidence rates are approximate categories, we instead report descriptive statistics on the CTS sum scores and gender differences therein using Mann-Whitney-U tests.

We estimated the direct and indirect effects of victimization on perpetration using moderated mediation (63). We investigated whether victimization had an effect on the outcome via two pathways: a direct association with perpetration of assault and an indirect effect via the construct of anger. It was further examined whether the indirect and/or direct anger pathways were moderated by gender. Continuous subscale scores were used for mild and severe victimization, perpetration and anger disposition. We used multivariable linear regression models for each of the pathways presented in **Figure 1**. Moderation effects were modelled with interaction terms, first starting with a full model including an interaction between gender and each type of victimization and then conducting model selection based on the Akaike information criterion (AIC) to find which interaction terms could be excluded. Models with the lowest AIC values were selected. We report on unstandardized coefficients throughout, except when comparing the associations between male and female subgroups where standardized coefficients are reported on. The analyses were conducted using R and the package “mediate” (version 4.5; Tingley et al. (64)). Estimating standard errors and p-values of mediation effects were done through quasi-Bayesian simulations (n = 500). Heteroscedasticity-consistent standard errors were used in the simulations to obtain robust estimates. The assumption of normality for the mediator was assessed by transforming the DAR scores via log, square root and Box-Cox transformations independently.

## Results

As shown in **Table 1**, the sample consisted mainly of men (63.5%). Mean age was 45 years, with women being approximately 3 years older than men. More than ¾ of the sample were diagnosed with a psychotic disorder. The remaining patients were diagnosed with major depressive disorder or bipolar disorder. Most patients were single. One out of five men were in a committed relationship compared to one in three women. Employment was rare. Most patients were of Dutch origin. Other ethnic groups were Turkish, Moroccan or Surinamese. The study sample was representative of the Dutch SMI patient population (58).

**Table 1:** Demographic characteristics of the respondents (N=942).

	Total (N=942)	Men (N=598, 63.5%))	Women (N=344, 36.5%))	Test statistic (df), significance
	N (valid %)	N (valid %)	N (valid %)	
Age (m;sd)	44.8 (10.4)	43.7 (10.3)	46.6 (10.4)	T (941)= -4.2, p <0.001***
Diagnosis				χ²(1)=31.1, p <0.001***
Psychotic disorders	728 (77.3)	496 (82.9)	232 (67.5)	
Other	212 (22.5) 214 (22.7)	102 (17.1)	112 (32.5)	
Marital status				χ²(1)=18.4, p= <0.001***
Single	708 (75.1)	477 (79.8)	231 (67.2)	
Committed relationship	234 (24.9)	121 (20.2)	113 (32.8)	
Employment				χ²(1)=5.7, p=0.02*
Yes	134 (14.2)	98 (16.4)	36 (10.5)	
No	808 (85.8)	500 (83.6)	308 (89.5)	
Ethnicity				χ²(1)=1.2, p=0.3
Dutch	583 (61.9)	362 (60.5)	221 (64.2)	
Other	359 (38.1)	236 (39.5)	123 (35.8)	
Educational level				χ²(1) 6.8, p= 0.009**
Low -.Mid	539 (56.8)	359 (60.0)	177 (51.5)	
Mid - High	407 (43.2)	239 (40.0)	167 (48.5)	

m, mean; sd, standard deviation; χ², chi-square; df, degrees of freedom; T, students t-test; *significance at the <0.05 level, **<0.01 level, ***<0.001 level, Low- Mid = no education-preparatory vocational education, Mid-High= intermediate vocational education-academic education.

The mean score on the DAR was 11.7 (SD: 5.0). Men did not differ from women significantly. Fifteen percent of the patients (145/942) scored in the range of pathological anger: 16% (98/500) of the male versus 14% of the female SMI patients (47/344) (X^2^(1)=1.05; p=0.298).

### Prevalence of perpetration and victimization

**Figure 2:**
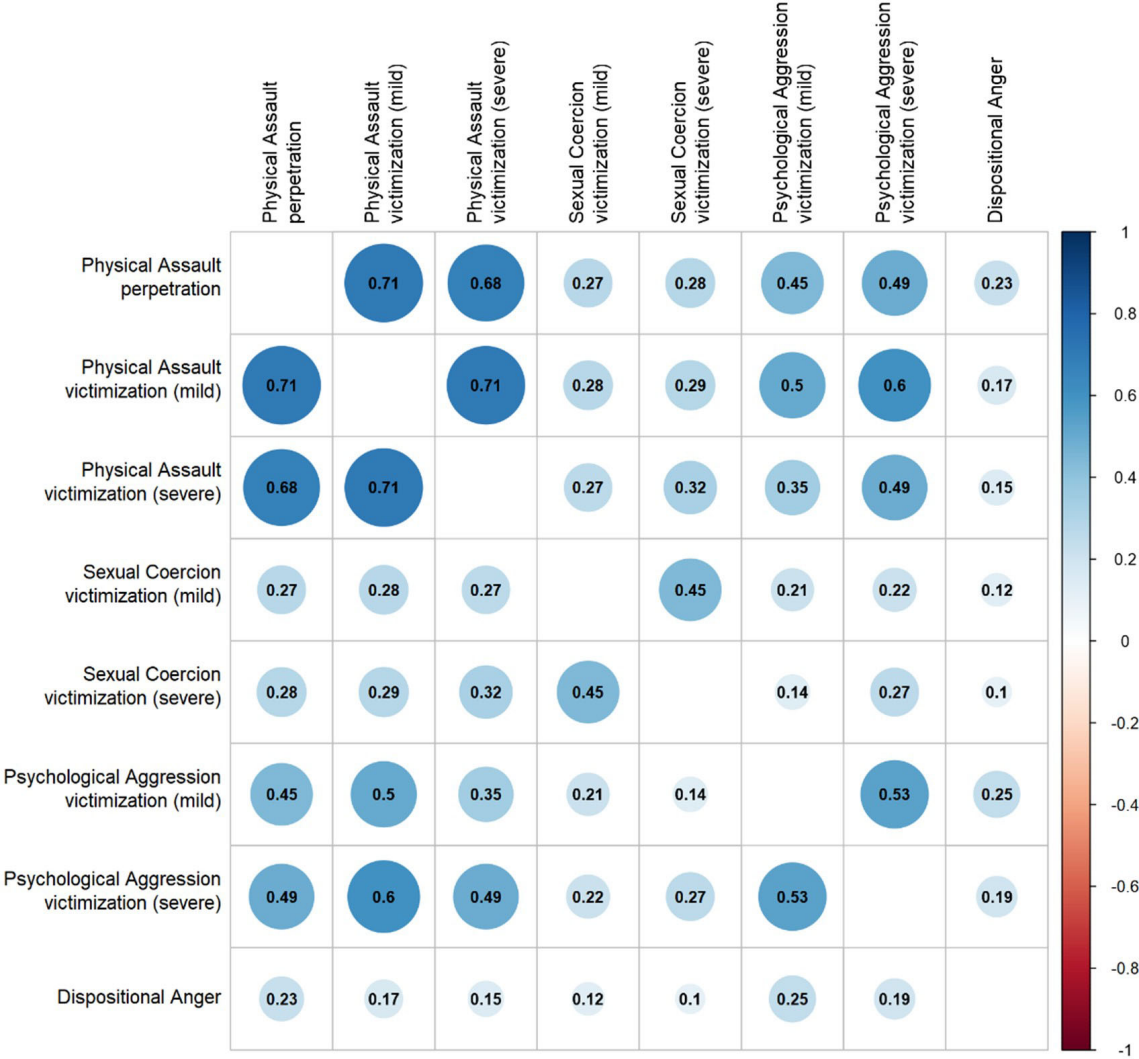
The correlations between the variables. *DAR, Dimensions of Anger Reactions.

**Table 2** reports the 12-month prevalence of mild and severe forms of perpetration and victimization. The number of incidences is depicted in **Supplementary Table 2**. Twenty two percent of the patients in the sample perpetrated physical assault. Twelve percent of the patients committed only mild forms and 8% committed both mild and severe forms. Men and women reported similar prevalence rates of mild and severe forms of perpetration. **Supplementary Table 2** shows the range of the number of incidents reported. As expected, the range of the number of minor incidents was larger than the number of severe incidents, with a maximum of 70 incidents over the past year among men. However, the majority reported five or fewer incidents in the past year. There was a strong correlation between perpetration of physical assault and victimization of physical assault (**Figure 2**). **Supplementary Table 3** shows that 52% of patients were both victim and perpetrator of physical assault.

**Table 2:** Prevalence of the perpetration and victimization in the context of DVA of physical assault, victimization of sexual coercion and psychological aggression, stratified by gender.

Conflicts Tactics Scale -2	Total % (N=942)	95%-CI	#Men % (N=598)	95%-CI	Women % (N=344)	95%-CI	OR (95%CI)
Perpetration							
Physical Assault							
Mild	12.4	10.3-14.6	12.9	10.2-15.6	11.6	8.2-15.0	1.0 (0.7-1.4)
Mild and severe	8.4	6.6-10.2	7.8	5.7-10.0	9.3	6.2-12.3	1.2 (0.7-1.9)
Severe	1.3	0.7-2.2	1.9	1.0-3.3	0.3	0.0-1.6	1.0 (0.6-1.6)
Total	22.1	19.4-24.7	22.6	19.2-25.9	21.2	16.9-25.5	0.9 (0.7-1.3)
Victimization							
Physical Assault							
Mild	12.9	10.8-15.1	11.0	8.5-13.5	16.3	12.4-20.2	1.3 (1.0-1.8)
Mild and severe	11.8	9.7-13.8	11.9	9.2-14.5	11.6	8.2-15.0	1.0 (0.7-1.5)
Severe	2.3	1.6-3.5	2.5	1.5-4.1	1.8	0.8-3.8	0.9 (0.5-1.7)
Total	27.0	24.1-29.8	25.4	21.9-28.9	29.7	24.8-34.4	1.2 (0.9-1.7)
Sexual Coercion							
Mild only	3.7	2.5-4.9	2.8	1.5-4.2	5.2	2.9-7.6	2.1 (1.2-3.7)*
Mild and severe	1.8	1.0-2.7	1.2	0.3-2.0	2.9	1.1-4.7	2.5 (1.0-6.7)
Severe	0.9	0.5-1.8	1.2	0.6-2.4	0.3	0.0-1.6	1.4 (0.7-3.1)
Total	6.4	4.8-7.9	5.2	3.4-7.0	8.4	5.5-11.4	1.7 (1.0-2.8)
Psychological aggression							
Mild only	29.4	26.5-32.3	29.8	26.1-33.4	28.8	24.0-33.7	1.1 (0.8-1.4)
Mild and severe	26.8	23.9-29.6	25.8	22.2-29.3	28.5	23.7-33.3	1.2 (0.9-1.6)
Severe	3.0	2.1-4.3	2.9	1.8-4.5	3.2	1.8-5.6	0.8 (0.4-1.4)
Total	59.2	56.1-62.3	58.5	54.6-62.5	60.5	55.3-65.6	1.1 (0.8-1.4)

CI, confidence interval; OR, odds ratio; *significant difference at the <0.05 level. #men are reference category.

A total of 27% of patients reported being a victim of any type of physical assault over the past year (**Table 2**). Thirteen percent were victims of mild forms only, but an equally large group of patients (12%) were victims of both mild and more severe forms of physical assault. Women reported slightly more victimization of both mild and severe forms, but these differences were not significant. However, the maximum number of reported incidents of physical assault is higher in women for both mild and severe forms of physical assault. This trend is more pronounced for sexual coercion. Severe forms remain rare (1-3% of patients), but the odds for women to report to be victims of sexual coercion is twice as high compared to the odds of men doing so (OR =2.1). Overall, 5% of men have been victims of some form of sexual coercion in the past year, compared with 8% of women. In line with this, incident rates show that women reported relatively more severe incidents (median: 7.75 incidents), compared to less severe incidents (median: 6.5 incidents), and more severe incidents compared to men (median: 3 incidents). Additionally, there were 2 female patients who reported having been sexually victimized approximately 50 times in the past year.

More than half of the patients (59.2%) have experienced one or more form of psychological aggression over the past year. For one third of the patients this were minor forms only. However, for a quarter of the patients these included severe forms as well. Again, we see more victimization in women than in men, but no significance.

### Moderated mediation analysis

To investigate whether the direct association between victimization and perpetration was mediated by anger and moderated by gender, we conducted a moderated mediation analysis using minor and severe as subscales of the three types of victimization as predictors. Gender was added as an interaction term in both the indirect and direct pathway model, allowing each of the six subscales of victimization-severity direct and indirect associations to vary by gender. Conducting AIC model selection on the indirect pathway model suggested that gender did not moderate the relationship between victimization and anger, as all interaction terms were insignificant, and their inclusion resulted in a worse model fit. The same AIC selection applied to the direct pathway model revealed three significant interactions terms that improved the model fit: gender with minor physical abuse (p = .004), with severe physical abuse (p <.001) and with severe psychological abuse (p = .012).

The moderated mediation analysis was conducted again using the best fitting models, i.e., the direct effect with the three significant interactions and the indirect effect with no interactions. All unstandardized direct, indirect and total effects are presented in **Table 3** before adding the gender interaction. Dispositional anger only mediated the link between minor psychological aggression (unstandardized beta = 0.02; CI = 0.01, 0.03; p <.001) and perpetration. Minor psychological aggression was also directly associated with DVA perpetration of physical assault (unstandardized beta = 0.10; CI = 0.05, 0.16; p <.001). We also found significant direct effects for both minor (unstandardized beta= 0.56; CI = 0.45, 0.65; p <.001) and severe physical assault (unstandardized beta = 0.61; CI = 0.49, 0.72; p <.001) on perpetration, in absence of mediation effects via anger.

**Table 3:** Unstandardized indirect, direct, and total effects before added moderated effects, in the mediation model for each type and severity of victimization on violent perpetration via anger as a mediator.

Effect	Estimate	CI Lower	CI Upper	p-value
Physical assault (mild)				
Indirect effect	-0.001	-0.014	0.011	0.780
Direct effect	0.555	0.454	0.647	<.001*
Total effect	0.554	0.452	0.646	<.001*
Physical assault (severe)				
Indirect effect	0.006	-0.007	0.021	0.42
Direct effect	0.608	0.493	0.731	<.001*
Total effect	0.613	0.498	0.739	<.001*
Sexual coercion (mild)				
Indirect effect	0.017	-0.004	0.046	0.14
Direct effect	0.142	-0.048	0.334	0.14
Total effect	0.160	-0.035	0.351	0.09
Sexual coercion (severe)				
Indirect effect	0.009	-0.015	0.038	0.51
Direct effect	0.141	-0.075	0.370	0.20
Total effect	0.150	-0.065	0.376	0.18
Psychological aggression (mild)				
Indirect effect	0.018	0.007	0.030	<.001*
Direct effect	0.103	0.045	0.160	<.001*
Total effect	0.121	0.061	0.177	<.001*
Psychological aggression (severe)				
Indirect effect	0.008	-0.003	0.024	0.19
Direct effect	0.026	-0.071	0.123	0.61
Total effect	0.034	-0.067	0.136	0.51

CI, 95% confidence interval; ** significance at p <0.01.

**Figure 3:**
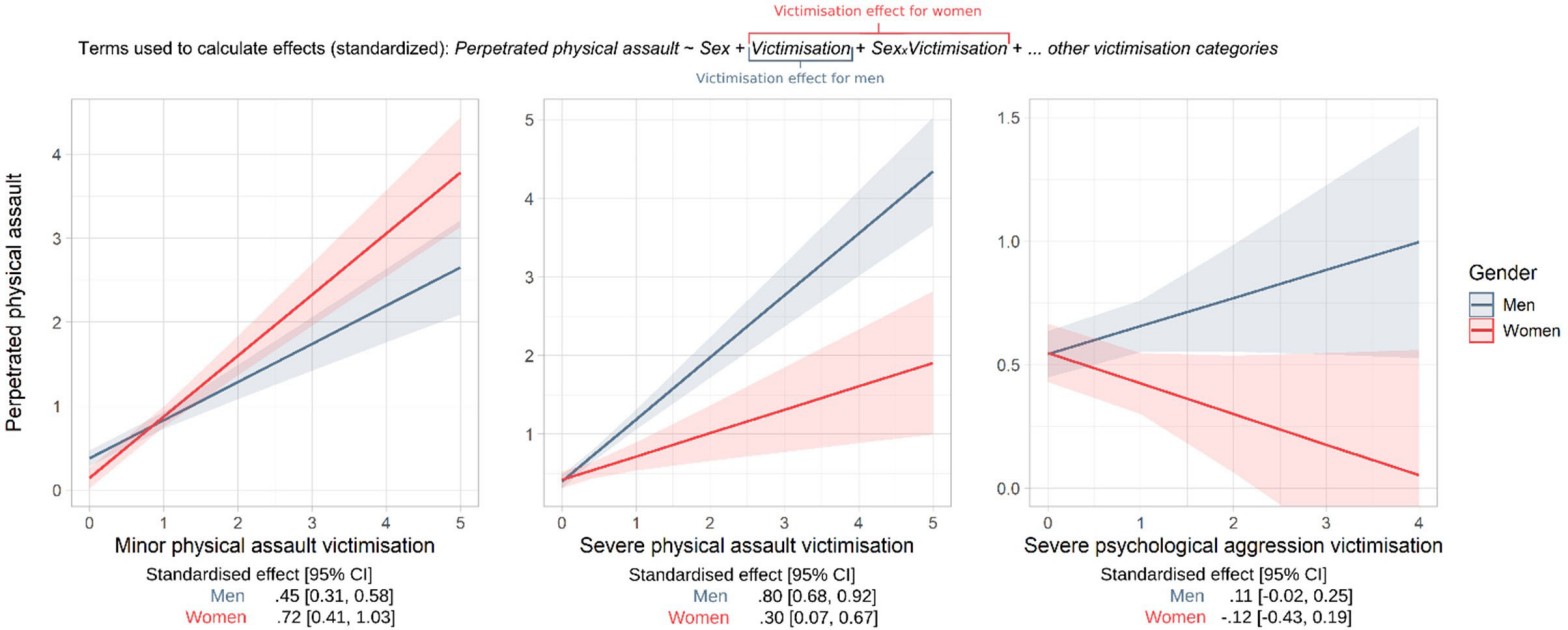
Sex differences in predicted assault perpetration presented as a function of physical and psychological victimization. CI, confidence interval. Lines correspond to estimated marginal means, shaded area to 95% confidence interval.

However, after testing for moderation, we found evidence of gender differences for these direct effects. Effect plots illustrating these interactions between genders are presented in **Figure 3**. This reveals that in the group of people being a victim of minor physical assault, the perpetration of physical assault in women was higher [standardized effect = .72 (95%CI = 0.41, 1.03)] than for men [0.45 (0.31, 0.58)], but in both genders, the relationship was pronounced positive. Conversely, perpetration was higher for men when severe physical victimization was also high. Again, the relationship was positive for both genders, but much stronger for men [0.80 (0.68, 0.92)] than for women [0.30 (0.07, 0.67)]. Men with more severe psychological victimization again had higher perpetration scores [0.11 (-0.02, 0.25)], while there was a negative association in women [-0.12 (-0.43, 0.19)]. Although these effects were smaller than the previously reported association, the diverging direction of this effect between men and women explains the significance of the interaction. Additionally, we found no associations between sexual coercion (minor nor severe) and victimization, anger, gender, and violent perpetration. As a sensitivity analysis, we transformed the skewed DAR sum score using a natural log, square root and Box-Cox transformation. The direction, effect size and significance of the results remained the same.

## Discussion

In this large sample of SMI patients, we found that one in five SMI patients were perpetrators of physical assault and more than one in four SMI patients was victims of physical assault in the context of DVA. A percentage of 52% of these patients were both victim and perpetrator of physical assault. A total of 6.4% of SMI patients were victims of sexual coercion and more than half were victims of psychological aggression in the past 12 months. Overall, anger had no mediating effect on the association between victimization and perpetration of violence except for the link between the victimization of mild psychological aggression and perpetration of physical assault. In addition, gender moderated the association between mild physical assault victimization and perpetration of physical assault.

We found high prevalence rates for victimization of physical assault in both men and women, and even higher prevalence rates for victimization of psychological aggression in both men and women. We saw no significant differences between men and women in prevalence of victimization or perpetration of DVA, which is similar to other studies (30, 31, 65). Another result was that about half of the patients who were victims of minor physical assault, were also victims of severe physical assault. This means that one in eight outpatients have been victims of severe physical assault like being chocked or confronted with a knife or gun in the past 12 months by a friend/roommate/partner. These offenses could result in injuries requiring medical care, and/or trauma. It is therefore noteworthy that detection of these cases by mental health professionals is still very low (25, 66). A previous systematic review reported a lower prevalence of victimization of physical violence (11%, 95% CI 8-15%) in SMI patients than our study (24.0%, 95% CI 24.1-29.8%) (Khalifeh et al., (21)). Even when these results were stratified to compare men and women, the systematic review found that women were significantly more likely to be victims of physical assault compared to men (21). We did not find this difference in our study. This may be due to the use of different instruments to measure DVA. Bergman and Ericsson (67) used the unrevised CTS in SMI patients and reported higher prevalence rates of both victimization as perpetration of violence compared to the general population. However, Bergman and Ericsson (67) used a small sample of 55 patients and only hospitalized patients. In this study, we obtained a much larger sample and also included outpatients. Studies using the CTS in the general population often reported a lower prevalence of victimization of violence compared to our results, but also often found no difference in prevalence between men and women (68, 69).

In our sample, 6.4% of all participants experienced some form of sexual coercion in the previous year. This is much higher than the prevalence of sexual assault in the past year in the general population (70), which is 2% for women and less than 1% for men. However, the prevalence rate in this study is largely consistent with previous literature based on SMI patients (25, 35, 37). Khalifeh et al. (21) reported an overall prevalence of sexual violence of 9.9%, which is slightly higher than the prevalence of 8.4% in women in our study. In contrast to our study, this analysis included sexual violence in the past year as well as experienced sexual violence in the past three years, which could explain the higher percentage for female SMI patients. Although the prevalence rates of sexual coercion were higher in women than in men in our sample, the prevalence of sexual coercion we found in male SMI patients is much higher than what is currently reported in the literature (21, 35). This result indicates that sexual coercion in men with SMI is even more widespread than originally thought and often goes unnoticed (35).

Relatively little research has been done on the prevalence of perpetration of physical assault among SMI patients. The studies that investigated it, reported a wide range of prevalence rates ranging from 2% to 50% (71). A more recent study of Kivisto et al. (34) reported an overall prevalence of perpetration of physical assault of 20.3% in the year following discharge of psychiatric patients. In contrast to the general population -where men are more likely to commit domestic violence- female SMI patients reported a prevalence of perpetration of 29.4% compared to 13.9% in men. The overall prevalence in our study is similar to that reported by Kivisto et al. (34). This could mean that the women included in our study could have a tendency to react aggressively compared to the general population. However, since this was a cross sectional study, this theory could not be tested.

DVA victimization and perpetration were strongly correlated in our sample, but their relationship was largely unaffected by anger, except when participants were victims of mild psychological aggression. This result could imply that when patients are taunted or provoked, aggression overrides violence-inhibiting forces and they can retaliate with physical force (41), and this may occur within the same relationship or in different relationships. The temporal ordering between victimization and perpetration suggested here only on a theoretical level. Due to the cross-sectional nature of our study, it could also be the case that perpetration of DVA accompanied by anger increases the risk of subsequent DVA victimization; only studies using repeated measures can untangle these temporal dynamics.

The absence of a mediating effect of anger in the association between victimization and perpetration of violence in SMI patients, except for victimization of psychological aggression is in line with results from previous research (72, 73). Sprunger et al. (72) tested if the association of victimization and perpetration of DVA in the general population was mediated through anger and did not find significant results. Walters et al. (73), also did not find an anger-mediated association between being a victim and perpetrator of bullying a general population sample. Other research pointed out that not anger, but impulsivity and a lack of control could be the linking pin between aggressive behavior and perpetration of DVA (46, 74). Impulsivity and aggression are also often observed to co-occur in patients suffering from SMI who need hospitalization due to mental illness (75, 76). The patients in our sample, however, were all receiving outpatient community mental healthcare, meaning that they were clinically stable enough for receiving outpatient care, and no hospitalization, at the time the survey was completed. Therefore, we could assume these patients had less clinical symptoms and may have showed less impulsive behavior than patients needing hospitalization due to their mental state. Also, patients suffering from SMI often are prescribed medication that can reduce impulsivity and aggression (77). This assumed decrease of impulsive behavior in the SMI patients included in our study sample combined with medication use could explain the fact that we did not find a mediating effect of anger in our study. However, testing this hypothesis is beyond the scope of this study as we do not have the data on medication use, level of impulsiveness and on mental stability in our study population.

Another explanation for the absence of a mediation effect of anger, could be that the quality of anger could play a role in the occurrence of violence and also the severity of violence (41, 52). Several studies suggested that a more provocative use of expressed anger such as contempt or hostility could result in aggression (78, 79). Our results seem to support this result because we did see anger acting as a mediator between perpetration of physical assault and victimization of psychological aggression. This would suggest that the quality of what is said during altercations affects if and how a person will reciprocate using physical aggression.

Previous studies showed that women in the general population tended to retaliate with violence for being hurt emotionally (80–82). This phenomenon was not yet studied in men. Interaction effects in this study revealed gender differences. Women were more likely to perpetrate violence if they are victims of minor physical assault compared to men. This supports the previous mentioned theory of retaliation when the balance between violence inhibiting forces and violence provoking forces is distorted (41, 83). However, the interaction effects also showed a negative link between severe victimization of psychological aggression and physical assault in women. This finding does not support the retaliation theory, but rather seems to reflect the effect of ‘learned helplessness’ (84). Learned helplessness is a psychological phenomenon, in which victims who are repeatedly exposed to aggressive stimuli (like violence) eventually will adapt a passive coping style and emotional numbness (84). Learned helplessness thus means that the more victims are exposed to these stimuli, the less often they will retaliate or react. The results in our study show that this phenomenon could partly explain the lower level of perpetration of violence by female SMI patients who are a victim of severe psychological aggression (**Figure 3**).

### Strengths and limitations

Strengths of this study included its large sample size of SMI patients and using established self-report instruments with good psychometric properties. The CTS-2 was originally developed to measure domestic violence in romantic relationships. Most participants in our sample were not involved in a romantic relationship. The survey was therefore adapted for participants who were not in a romantic relationship.

Limitations were that the precise nature of the violence and the relationship of the victim/perpetrator with the respective participant remain unclear. Similarly, we have no information on the extent to which the violent interactions happened in the context of one or more specific relationships. However, because of the way the questions in the CTS-2 are phrased, we can safely assume that the perpetrator or victim is someone the participant knows. Also, because the CTS-2 measures more subtle forms of violence such as psychological abuse, we assume that the estimation of both perpetration and victimization of these forms is more accurate than estimates based on police data since a smaller percentage of victims seek formal support (85, 86).

Another limitation of the study includes the cross-sectional design, making it impossible to test the directionality and the causality of effects. In addition, we did not obtain questions on perpetration of sexual coercion and psychological aggression.

Moreover, the context in which the violence occurred was not known in our study. Unfortunately we do not have data on in which circumstances victimization or perpetration of violence happened. Circumstances like intoxication with alcohol, or active psychotic symptoms could not be measured. However, severe symptomatology, high levels of aggression or cognitive impairments were exclusion criteria. Also, already mentioned earlier, violence in the context of threatening behavior could be triggered by different mechanisms than systematic violence without a known trigger. Johnson et al. suggests in his research that the CTS does not measure the difference between coercive controlling violence and situational couple violence (87). However, at the time this study was conducted, there were no reliable surveys to assess coercive control (88). Also, the DAR survey used to measure anger might not be adequate and/or specific enough to differentiate between level of anger and actual aggression, which could have a different effect on the relationship between perpetration and victimization of physical assault. However, the DAR survey was compared with other surveys on anger and found to be efficient (61), as was the revised CTS used (59, 60, 89).

## Clinical implications and conclusion

This study showed relatively high prevalence rates of victimization and perpetration of DVA in patients with SMI. While the focus has been mostly on female victimization, our study shows that men should not be overlooked. We found no difference between men and women in rates of victimization and perpetration of physical violence and no difference in prevalence rates of victimization of psychological aggression and sexual coercion either. We also showed that the pathway between victimization and perpetration of physical assault was not mediated by anger, except for the link between minor psychological aggression victimization and perpetration of physical violence. This could imply that anger is specifically relevant in explaining physical aggression among individuals who have been victims of psychological aggression, but not in other forms of aggression that perhaps trigger different emotional sequelae (e.g., shame, fear). We did see that women tend to report a higher prevalence of minor sexual coercion. However, we suspect an under detection in male SMI patients, because of the taboo on victimization of sexual assault in men (35).

Since we did not find a single underlying mechanism to explain victimization and perpetration of DVA, but different mechanisms affecting each other, this study underlines the complexity of DVA and further supports that the mechanisms of DVA lie in risk heterogeneity. Our study emphasizes the importance of disclosure of violence and to make it a standard topic during mental health care sessions. It should be mandatory to talk to every patient about violence on a regular basis, particularly because being a victim of violence can have severe consequences for both mental and physical health and because patients who are a victim of violence are likely to be a perpetrator as well. Clinicians should also be aware of the context in which the violence occurs in order to be able to provide personalized care to help and assist a potential victim of perpetrator to better their situation. Future research should focus on mechanisms underlying this cycle of violence in their context and should develop effective interventions to prevent and reduce violence in SMI patients (90).

## Data Availability

The raw data supporting the conclusions of this article will be made available by the authors, without undue reservation.

## References

[1] World Health Organizaion. (2024). Available online at: https://apps.who.int/violence-info/intimate-partner-violence/ (Accessed May 11, 2024).

[2] Stuurgroep multidisciplinaire Richtlijnontwikkeling in de GGZ. Richtlijn: familiaal huiselijk geweld. In: Guideline: domestic violence within families. Trimbos Instituut, Utrecht (2009).

[3] Sardinha L Maheu-Giroux M Stöckl H Meyer SR García-Moreno C. Global, regional, and national prevalence estimates of physical or sexual, or both, intimate partner violence against women in 2018. Lancet (London England). (2022) 399:803–13. doi: 10.1016/S0140-6736(21)02664-7 PMC8885817 35182472

[4] World Health Organization. Global and regional estimates of violence against women. In: Prevalence and health effects of intimate partner violence and non-partner sexual violence (2013) (Geneva, Switzerland: The World Health Organization).

[5] Spencer CN Khalil M Herbert M Aravkin AY Arrieta A Baeza MJ. Health effects associated with exposure to intimate partner violence against women and childhood sexual abuse: a Burden of Proof study. Nat Med. (2023) 29:3243–58. doi: 10.1038/s41591-023-02629-5 PMC10719101 38081957

[6] Ojong SA Temmerman M Khosla R Bustreo F. Women’s health and rights in the twenty-first century. Nat Med. (2024) 30:1547–55. doi: 10.1038/s41591-024-03036-0 38886622

[7] Breiding MJ Black MC Ryan GW. Chronic disease and health risk behaviors associated with intimate partner violence-18 U.S. states/territories, 2005. Ann Epidemiol. (2008) 18:538–44. doi: 10.1016/j.annepidem.2008.02.005 18495490

[8] Wong J Mellor D. Intimate partner violence and women’s health and wellbeing: impacts, risk factors and responses. Contemp Nurse. (2014) 46:170–9. doi: 10.5172/conu.2014.46.2.170 24787250

[9] Gibson CJ Bahorik A Xia F Peltz C Yaffe K. Intimate partner violence, mental health, and aging-related health among men and women veterans across the lifespan. J Gen Intern Med. (2024) 39:931–9. doi: 10.1007/s11606-023-08466-z PMC11074085 37962725

[10] Nguyen KA Abrahams N Jewkes R Mhlongo S Seedat S Myers B. The associations of intimate partner violence and non-partner sexual violence with hypertension in South African women. Int J Environ Res Public Health. (2022) 19:4026. doi: 10.3390/ijerph19074026 35409715 PMC8998257

[11] Lagdon S Armour C Stringer M. Adult experience of mental health outcomes as a result of intimate partner violence victimisation: a systematic review. Eur J Psychotraumatol. (2014) 12:5. doi: 10.3402/ejpt.v5.24794 PMC4163751 25279103

[12] Loxton D Dolja-Gore X Anderson AE Townsend N. Intimate partner violence adversely impacts health over 16 years and across generations: A longitudinal cohort study. PloS One. (2017) 12:e0178138. doi: 10.1371/journal.pone.0178138 28582406 PMC5459340

[13] Daugherty JC Marañón-Murcia M Hidalgo-Ruzzante N Bueso-Izquierdo N Jiménez-González P Gómez-Medialdea P. Severity of neurocognitive impairment in women who have experienced intimate partner violence in Spain. J Forensic Psychiatry Psychol. (2019) 30:322–40. doi: 10.1080/14789949.2018.1546886

[14] Ferrari G Agnew-Davies R Bailey J Howard L Howarth E Peters TJ. Domestic violence and mental health: a cross-sectional survey of women seeking help from domestic violence support services. Glob Health Action. (2016) 9:29890. doi: 10.3402/gha.v9.29890 26860876 PMC4748088

[15] Sparrow K Kwan J Howard L Fear N MacManus D. Systematic review of mental health disorders and intimate partner violence victimisation among military populations. Soc Psychiatry Psychiatr Epidemiol. (2017) 52:1059–80. doi: 10.1007/s00127-017-1423-8 PMC5581819 28748307

[16] Sesar K Dodaj A Simic N. Mental health of perpetrators of intimate partner violence. Ment Health Rev J. (2018) 23:221–39. doi: 10.1108/MHRJ-08-2017-0028

[17] Shorey RC Febres J Brasfield H Stuart GL. The prevalence of mental health problems in men arrested for domestic violence. J Fam Violence. (2012) 27:741–8. doi: 10.1007/s10896-012-9463-z PMC3532855 23284227

[18] Ramsoomar L Gibbs A Chirwa ED Machisa MT Alangea DO Addo-Lartey AA. Pooled analysis of the association between mental health and violence against women: evidence from five settings in the Global South. BMJ Open. (2023) 13:e063730. doi: 10.1136/bmjopen-2022-063730 PMC10030569 36921941

[19] Breet E Seedat S Kagee A. Posttraumatic stress disorder and depression in men and women who perpetrate intimate partner violence. J Interpers Violence. (2019) 34:2181–98. doi: 10.1177/0886260516660297 27432455

[20] Devries KM Mak JY Bacchus LJ Child JC Falder G Petzold M. Intimate partner violence and incident depressive symptoms and suicide attempts: A systematic review of longitudinal studies. PLoS Med. (2013) 10:e1001439. doi: 10.1371/journal.pmed.1001439 23671407 PMC3646718

[21] Khalifeh H Oram S Osborn D Howard LM Johnson S. Recent physical and sexual violence against adults with severe mental illness: a systematic review and meta-analysis. Int Rev Psychiatry. (2016) 28:433–51. doi: 10.1080/09540261.2016.1223608 PMC5309869 27645197

[22] Oram S Khalifeh H Howard LM. Violence against women and mental health. Lancet Psychiatry. (2017) 4:159–70. doi: 10.1016/S2215-0366(16)30261-9 27856393

[23] Oram S Trevillion K Feder G Howard LM. Prevalence of experiences of domestic violence among psychiatric patients: Systematic review. Br J Psychiatry. (2013) 202:94–9. doi: 10.1192/bjp.bp.112.109934 23377208

[24] White SJ Sin J Sweeney A Salisbury T Wahlich C Montesinos Guevara CM. Global prevalence and mental health outcomes of intimate partner violence among women: A systematic review and meta-analysis. Trauma Violence Abuse. (2024) 25:494–511. doi: 10.1177/15248380231155529 36825800 PMC10666489

[25] Trevillion K Oram S Feder G Howard LM. Experiences of domestic violence and mental disorders: a systematic review and meta-analysis. PLoS One. (2012) 7:e51740. doi: 10.1371/journal.pone.0051740 23300562 PMC3530507

[26] Yu R Nevado-Holgado AJ Molero Y D’Onofrio BM Larsson H Howard LM. Mental disorders and intimate partner violence perpetrated by men towards women: A Swedish population-based longitudinal study. PLoS Med. (2019) 16:e1002995. doi: 10.1371/journal.pmed.1002995 31846461 PMC6917212

[27] Whiting D Gulati G Geddes JR Fazel S. Association of schizophrenia spectrum disorders and violence perpetration in adults and adolescents from 15 countries: A systematic review and meta-analysis. JAMA Psychiatry. (2022) 79:120–32. doi: 10.1001/jamapsychiatry.2021.3721 PMC8696689 34935869

[28] Spencer CM Keilholtz BM Palmer M Vail SL. Mental and physical health correlates for emotional intimate partner violence perpetration and victimization: A meta-analysis. Trauma Violence Abuse. (2024) 25:41–53. doi: 10.1177/15248380221137686 36458866

[29] Okuda M Olfson M Wang S Rubio JM Xu Y Blanco C. Correlates of intimate partner violence perpetration: results from a National Epidemiologic Survey. J Trauma Stress. (2015) 28:49–56. doi: 10.1002/jts.2015.28.issue-1 25624189

[30] Ansara DL Hindin MJ. Perpetration of intimate partner aggression by men and women in the Philippines: prevalence and associated factors. J Interpers Violence. (2009) 24:1579–90. doi: 10.1177/0886260508323660 18768743

[31] Straus MA. Dominance and symmetry in partner violence by male and female university students in 32 nations. Children Youth Serv Review. (2008) 30:252–75. doi: 10.1016/j.childyouth.2007.10.004

[32] MaChado A Sousa C Cunha O. Bidirectional violence in intimate relationships: A systematic review. Trauma Violence Abuse. (2024) 25:1680–94. doi: 10.1177/15248380231193440 37594220

[33] Thornton AJ Graham-Kevan N Archer J. Intimate partner violence: Are the risk factors similar for men and women, and similar to other types of offending? Aggress Behav. (2016) 42:404–12. doi: 10.1002/ab.21635 26678658

[34] Kivisto AJ Watson ME. 12-month prevalence, trends, gender differences, and the impact of mental health services on intimate partner violence perpetration among discharged psychiatric inpatients. J Fam Viol. (2016) 31:379–85. doi: 10.1007/s10896-015-9780-0

[35] Zarchev M Ruijne RE Mulder CL Kamperman AM. Prevalence of adult sexual abuse in men with mental illness: Bayesian meta-analysis. BJPsych Open. (2021) 8:e16. doi: 10.1192/bjo.2021.1069 34915966 PMC8715257

[36] World Health Organization. Violence against women prevalence estimates, 2018: global, regional and national prevalence estimates for intimate partner violence against women and global and regional prevalence estimates for non-partner sexual violence against women. Geneva: World Health Organization (2021).

[37] Kamperman AM Henrichs J Bogaerts S Lesaffre EM Wierdsma AI Ghauharali RR. Criminal victimisation in people with severe mental illness: a multi-site prevalence and incidence survey in the Netherlands. PLoS One. (2014) 9:e91029. doi: 10.1371/journal.pone.0091029 24609108 PMC3946683

[38] Turanovic JJ. Heterogeneous effects of adolescent violent victimization on problematic outcomes in early adulthood. Criminology. (2019) 57:105–35. doi: 10.1111/crim.2019.57.issue-1

[39] Clare CA Velasquez G Mujica Martorell GM Fernandez D Dinh J Montague A. Risk factors for male perpetration of intimate partner violence: A review. Aggression Violent Behavior. (2021) 56:101532. doi: 10.1016/j.avb.2020.101532

[40] Hine B Bates EA Mackay J Graham-Kevan N. Comparing the demographic characteristics, and reported abuse type, contexts and outcomes of help-seeking heterosexual male and female victims of domestic violence: part I – who presents to specialist services? Partner Abuse. (2022) 1:20–60. doi: 10.1891/PA-2021-0009

[41] Birkley EL Eckhardt CI. Anger, hostility, internalizing negative emotions, and intimate partner violence perpetration: A meta-analytic review. Clin Psychol Rev. (2015) 37:40–56. doi: 10.1016/j.cpr.2015.01.002 25752947 PMC4385442

[42] Elkins SR Moore TM McNulty JK Kivisto AJ Handsel VA. Electronic diary assessment of the temporal association between proximal anger and intimate partner violence perpetration. Psychol Violence. (2013) 3:100–13. doi: 10.1037/a0029927

[43] Swan SC Gambone LJ Caldwell JE Sullivan TP Snow DL. A review of research on women’s use of violence with male intimate partners. Violence Vict. (2008) 23:301–14. doi: 10.1891/0886-6708.23.3.301 PMC2968709 18624096

[44] Smedslund J. How shall the concept of anger be defined? Theory Psychol. (1993) 3:5–33. doi: 10.1177/0959354393031001

[45] Fernandez E Johnson SL. Anger in psychological disorders: Prevalence, presentation, etiology and prognostic implications. Clin Psychol Rev. (2016) 46:124–35. doi: 10.1016/j.cpr.2016.04.012 27188635

[46] Shorey RC Sherman AE Kivisto AJ Elkins SR Rhatigan DL Moore TM. Gender differences in depression and anxiety among victims of intimate partner violence: The moderating effect of shame proneness. J Interpers Violence. (2011) 26:1834–50. doi: 10.1177/0886260510372949 20587460

[47] Charak R Byllesby BM Roley ME Claycomb MA Durham TA Ross J. Latent classes of childhood poly-victimization and associations with suicidal behavior among adult trauma victims: Moderating role of anger. Child Abuse Negl. (2016) 62:19–28. doi: 10.1016/j.chiabu.2016.10.010 27780110

[48] Twardawski M Angerl EM Lobbestael J. The effect of aggressive fantasizing on aggressive inclinations: Moderating effects of dispositional anger expression. Aggress Behav. (2024) 50:e22143. doi: 10.1002/ab.22143 38468496

[49] Norlander B Eckhardt C. Anger, hostility, and male perpetrators of intimate partner violence: a meta-analytic review. Clin Psychol Rev. (2005) 25:119–52. doi: 10.1016/j.cpr.2004.10.001 15642644

[50] Reagu S Jones R Kumari V Taylor PJ. Angry affect and violence in the context of a psychotic illness: a systematic review and meta-analysis of the literature. Schizophr Res. (2013) 146:46–52. doi: 10.1016/j.schres.2013.01.024 23452505

[51] Kuijpers KF van der Knaap LM Winkel FW. Risk of revictimization of intimate partner violence: the role of attachment, anger and violent behavior of the victim. J Fam Violence. (2012) 27:33–44. doi: 10.1007/s10896-011-9399-8 22389553 PMC3280382

[52] Neal AM Edwards KM. Perpetrators’ and victims’ Attributions for IPV: A critical review of the literature. Trauma Violence Abuse. (2017) 18:239–67. doi: 10.1177/1524838015603551 26346749

[53] Mauritz MW Goossens PJ Draijer N van Achterberg T. Prevalence of interpersonal trauma exposure and trauma-related disorders in severe mental illness. Eur J Psychotraumatol. (2013) 4. doi: 10.3402/ejpt.v4i0.19985 PMC3621904 23577228

[54] Rafiq S Campodonico C Varese F. The relationship between childhood adversities and dissociation in severe mental illness: a meta-analytic review. Acta Psychiatr Scand. (2018) 138:509–25. doi: 10.1111/acps.12969 30338524

[55] Iverson KM Dick A McLaughlin KA Smith BN Bell ME Gerber MR. Exposure to interpersonal violence and its associations with psychiatric morbidity in a U.S. national sample: A gender comparison. Psychol Violence. (2012) 3:273–87. doi: 10.1037/a0030956 PMC4163926 25232484

[56] Fulu E Miedema S Roselli T McCook S Chan KL Haardörfer R. Pathways between childhood trauma, intimate partner violence, and harsh parenting: findings from the UN Multi-country Study on Men and Violence in Asia and the Pacific. Lancet Glob Health. (2017) 5:e512–22. doi: 10.1016/S2214-109X(17)30103-1 28395846

[57] Fahlgren MK Cheung JC Ciesinski NK McCloskey MS Coccaro EF. Gender differences in the relationship between anger and aggressive behavior. J Interpers Violence. (2022) 37:NP12661–NP12670. doi: 10.1177/0886260521991870 33546562

[58] Delespaul PH de consensusgroep EPA. Consensus over de definitie van mensen met een ernstige psychische aandoening (EPA) en hun aantal in Nederland [Consensus regarding the definition of persons with severe mental illness and the number of such persons in the Netherlands. Tijdschr Psychiatr. (2013) 55:427–38. 23864410

[59] Straus MA Hamby SL Boney-McCoy SUE Sugarman DB. The revised conflict tactics scales (CTS2): development and preliminary psychometric data. J Family Issues. (1996) 17:283–316. doi: 10.1177/019251396017003001

[60] Chapman H Gillespie SM. The Revised Conflict Tactics Scales (CTS2): A review of the properties, reliability, and validity of the CTS2 as a measure of partner abuse in community and clinical samples. Aggress Viol Behav. (2019) 44:27–35. doi: 10.1016/j.avb.2018.10.006

[61] Forbes D Alkemade N Mitchell D Elhai JD McHugh T Bates G. Utility of the Dimensions of Anger Reactions-5 (DAR-5) scale as a brief anger measure. Depress Anxiety. (2014) 31:166–73. doi: 10.1002/da.2014.31.issue-2 23801571

[62] Nederlof AF Hovens JE Muris P Novaco RW. Psychometric evaluation of a Dutch version of the Dimensions of Anger Reactions. Psychol Rep. (2009) 105:585–92. doi: 10.2466/PR0.105.2.585-592 19928620

[63] Enders CK Hayes T Du H. A comparison of multilevel imputation schemes for random coefficient models: fully conditional specification and joint model imputation with random covariance matrices. Multivariate Behav Res. (2018) 53:695–713. doi: 10.1080/00273171.2018.1477040 30693802

[64] Tingley D Yamamoto T Hirose K Keele L Imai K. Mediation package for causal mediation analysis. J Stat Software. (2014) 59:1–38. doi: 10.18637/jss.v059.i05

[65] Kivisto AJ. Violence risk assessment and management in outpatient clinical practice. J Clin Psychol. (2016) 72:329–49. doi: 10.1002/jclp.2016.72.issue-4 26613557

[66] Ruijne R Mulder C Zarchev M Trevillion K van Est R Leeman E. Detection of domestic violence and abuse by community mental health teams using the BRAVE intervention: A multicenter, cluster randomized controlled trial. J Interpers Violence. (2022) 37:NP14310–NP14336. doi: 10.1177/08862605211004177 33866860 PMC9382347

[67] Bergman B Ericsson E. Family violence among psychiatric in-patients as measured by the Conflict Tactics Scale (CTS). Acta Psychiatr Scand. (1996) 94:168–74. doi: 10.1111/j.1600-0447.1996.tb09843.x 8891082

[68] Costa D Hatzidimitriadou E Ioannidi-Kapolou E Lindert J Soares J Sundin O. Intimate partner violence and health-related quality of life in European men and women: findings from the DOVE study. Qual Life Res. (2015) 24:463–71. doi: 10.1007/s11136-014-0766-9 25063083

[69] Lovestad S Krantz G. Men’s and women’s exposure and perpetration of partner violence: an epidemiological study from Sweden. BMC Public Health. (2012) 12:945. doi: 10.1186/1471-2458-12-945 23116238 PMC3534228

[70] de Haas S van Berlo W Bakker F Vanwesenbeeck I. Prevalence and characteristics of sexual violence in the Netherlands, the risk of revictimization and pregnancy: results from a national population survey. Violence Vict. (2012) 27:592–608. doi: 10.1891/0886-6708.27.4.592 22978077

[71] Choe JY Teplin LA Abram KM. Perpetration of violence, violent victimization, and severe mental illness: Balancing public health concerns. Psychiatr Serv. (2008) 59:153–64. doi: 10.1176/ps.2008.59.2.153 PMC3756896 18245157

[72] Sprunger JG Eckhardt CI Parrott DJ. Anger, problematic alcohol use, and intimate partner violence victimisation and perpetration. Criminal Behav Ment Health. (2015) 25:273–86. doi: 10.1002/cbm.v25.4 PMC4795827 26482016

[73] Walters GD Espelage DL. From victim to victimizer: Hostility, anger, and depression as mediators of the bullying victimization-bullying perpetration association. J Sch Psychol. (2018) 68:73–83. doi: 10.1016/j.jsp.2017.12.003 29861032

[74] Ammerman BA Kleiman EM Uyeji LL Knorr AC McCloskey MS. Suicidal and violent behavior: The role of anger, emotion dysregulation, and impulsivity. Pers Individ Dif. (2015) 79:57–62. doi: 10.1016/j.paid.2015.01.044

[75] Bousardt AMC Hoogendoorn AW Noorthoorn EO Hummelen JW Nijman HLI. Predicting inpatient aggression by self-reported impulsivity in forensic psychiatric patients. Crim Behav Ment Health. (2015) 26:161–73. doi: 10.1002/cbm.1955 25881695

[76] McDermott BE Holoyda BJ. Assessment of aggression in inpatient settings. CNS Spectrums. (2014) 19:425–31. doi: 10.1017/S1092852914000224 25296966

[77] van Schalkwyk GI Beyer C Johnson J Deal M Bloch MH. Antipsychotics for aggression in adults: A meta-analysis. Prog Neuropsychopharmacol Biol Psychiatry. (2018) 81:452–8. doi: 10.1016/j.pnpbp.2017.07.019 28754408

[78] Sommer J Iyican S Babcock J. The relation between contempt, anger, and intimate partner violence: A dyadic approach. J Interpers Violence. (2019) 34:3059–79. doi: 10.1177/0886260516665107 27543300

[79] Jacobson NS Gottman JM Waltz J Rushe R Babcock J Holtzworth-Munroe A. Affect, verbal content, and psychophysiology in the arguments of couples with a violent husband. J Consult Clin Psychol. (1994) 62:982–8. doi: 10.1037//0022-006x.62.5.982 7806730

[80] Leisring PA. Physical and emotional abuse in romantic relationships: motivation for perpetration among college women. J Interpers Violence. (2013) 28:1437–54. doi: 10.1177/0886260512468236 23262827

[81] Stuart GL Moore TM Gordon KC Ramsey SE Kahler CW. Psychopathology in women arrested for domestic violence. J Interpers Violence. (2006) 21:376–89. doi: 10.1177/0886260505282888 16443597

[82] Gupta G Sachdeva A Kumar M Singh M. Spectrum of intimate partner violence in patients with psychiatric illness-From victimization to perpetration. Int J Psychiatry Med. (2023) 58:20–36. doi: 10.1177/00912174211053726 35048727

[83] Finkel EJ. Impelling and inhibiting forces in the perpetration of intimate partner violence. Rev Gen Psychol. (2007) 11:193–207. doi: 10.1037/1089-2680.11.2.193

[84] Maier SF Seligman MEP. Learned helplessness - theory and evidence. J Exp Psychology-General. (1976) 105:3–46. doi: 10.1037/0096-3445.105.1.3

[85] Cavanaugh CE Martins SS Petras H Campbell JC. Mental disorders associated with subpopulations of women affected by violence and abuse. J Trauma Stress. (2013) 26:459–66. doi: 10.1002/jts.2013.26.issue-4 PMC4424795 23813596

[86] West CM Kantor GK Jasinski JL. Sociodemographic predictors and cultural barriers to help-seeking behavior by Latina and Anglo American battered women. Violence Vict. (1998) 13:361–75. doi: 10.1891/0886-6708.13.4.361 10328444

[87] Johnson MP Leone JM Xu Y. Intimate terrorism and situational couple violence in general surveys: ex-spouses required. Violence Against Women. (2014) 20:186–207. doi: 10.1177/1077801214521324 24504325

[88] Hardesty JL Crossman KA Haselschwerdt ML Raffaelli M Ogolsky BG Johnson MP. Toward a standard approach to operationalizing coercive control and classifying violence types. J Marriage Fam. (2015) 77:833–43. doi: 10.1111/jomf.12201 PMC4553695 26339101

[89] Straus MA Douglas EM. A short form of the Revised Conflict Tactics Scales, and typologies for severity and mutuality. Violence Vict. (2004) 19:507–20. doi: 10.1891/vivi.19.5.507.63686 15844722

[90] Ruijne RE Howard LM Trevillion K Jongejan FE Garofalo C Bogaerts S. Detection of domestic violence by community mental health teams: a multi-center, cluster randomized controlled trial. BMC Psychiatry. (2017) 17:288. doi: 10.1186/s12888-017-1399-7 28784096 PMC5545838

