# Supplementary material for: Anger’s moderating influence on the relationship between victimization and perpetration of domestic violence and abuse in patients suffering from severe mental illness. Insights from a cross sectional study using moderated mediation analysis
Source: Front Psychiatry. 2024 Dec 24;15:1509982. doi: 10.3389/fpsyt.2024.1509982 (PMC11704492; doi:10.3389/fpsyt.2024.1509982)
Supplement: Supplementary file 1 [file DataSheet1.docx]

**Supplementary Figure 1.** *Flowchart of the study sample.*

Random Sample

Invited

6%

8%

23%

Interview

Refused by mail

3336

No

77%

75%

92%

Experienced perpetration/victimization of domestic violence and abuse

942

1000

1763

58

763

162

764

647

1925

2572

Yeses

No

Missing or invalid data

**Reasons for non-response:**

No time/not interested/too tired: 81%

Failed appointments: 7%

No experience of victimization: 6%

Too traumatized: 2%

Other: 4%

**Reasons for non-response:**

No time/not interested/too tired: 84%

Failed appointments: 7%

No experience of victimization: 2%

Too traumatized: 5%

Other: 2%

Rejected

No

No

Yes

Yes

Yes

25%

43%

Approved

Contact

94%

57%%

Data cleaning

| **Supplementary table 2.** *Number of incidences of perpetration and victimization in the context of DVA of physical assault, victimization of sexual coercion, and psychological aggression, stratified by gender.* | | | |
| --- | --- | --- | --- |
|  | **Men** | **Women** | **p-value***^2^* |
|  | N= 608*^1^* | N= 348*^1^* |  |
| **Perpetration** |  |  |  |
| Physical assault |  |  |  |
| Mild | 1.23 (0.00, 70.00) | 1.04 (0.00, 36.00) | 0,9 |
| Severe | 0.44 (0.00, 40.00) | 0.36 (0.00, 15.00) | 0,9 |
| **Victimization** |  |  |  |
| Physical assault |  |  |  |
| Mild | 1.32 (0.00, 83.00) | 1.46 (0.00, 37.00) | 0,1 |
| Severe | 0.95 (0.00, 75.00) | 0.88 (0.00, 39.00) | >0.9 |
| Sexual aggression |  |  |  |
| Mild | 0.25 (0.00, 26.00) | 0.67 (0.00, 50.00) | 0,008* |
| Severe | 0.09 (0.00, 15.00) | 0.21 (0.00, 24.00) | 0,3 |
| Psychological aggression |  |  |  |
| Mild | 9.19 (0.00, 100.00) | 10.26 (0.00, 100.00) | 0,5 |
| Severe | 1.91 (0.00, 51.00) | 2.78 (0.00, 100.00) | 0,2 |
| *^1^*Mean (Range), *^2^*Mann-Whitney test, * significant at <0.001 level | | | |

|  | | | |  |
| --- | --- | --- | --- | --- |
| **Supplementary table 3.** Percentages of perpetrators of physical assault, victims of physical assault, psychological aggression or sexual coercion only and victims and perpetrators. | | | | |
|  | Perpetrator and victim | Perpetrator only | Victim only |  |
| **Psychological aggression** | 196 (34%) | 12 (2%) | 362 (64%) |  |
| **Sexual coercion** | 40 (18%) | 168 (74%) | 20 (9%) |  |
| **Physical assault (victim)** | 158 (52%) | 50 (16%) | 97 (32%) |  |
|  |  |  |  |  |
